# Urinary Eicosanoid Metabolites in HIV-Infected Women with Central Obesity Switching to Raltegravir: An Analysis from the Women, Integrase, and Fat Accumulation Trial

**DOI:** 10.1155/2014/803095

**Published:** 2014-06-01

**Authors:** Todd Hulgan, M. Sean Boger, Diana H. Liao, Grace A. McComsey, Christine A. Wanke, Alexandra Mangili, Sharon L. Walmsley, Heather McCreath, Ginger L. Milne, Stephanie C. Sanchez, Judith S. Currier, Jordan E. Lake

**Affiliations:** ^1^Vanderbilt University School of Medicine, Nashville, TN 37232, USA; ^2^Medical University of South Carolina, Charleston, SC 29403, USA; ^3^University of California, Los Angeles, CA 90035, USA; ^4^Case Western Reserve University, Cleveland, OH 44106, USA; ^5^Tufts University, Boston, MA 02111, USA; ^6^University of Toronto, Toronto, ON, Canada M5R 0A3

## Abstract

Chronic inflammation is a hallmark of HIV infection. Eicosanoids reflect inflammation, oxidant stress, and vascular health and vary by sex and metabolic parameters. Raltegravir (RAL) is an HIV-1 integrase inhibitor that may have limited metabolic effects. We assessed urinary F_2_-isoprostanes (F_2_-IsoPs), prostaglandin E_2_ (PGE-M), prostacyclin (PGI-M), and thromboxane B_2_ (TxB_2_) in HIV-infected women switching to RAL-containing antiretroviral therapy (ART). Thirty-seven women (RAL = 17; PI/NNRTI = 20) with a median age of 43 years and BMI 32 kg/m^2^ completed week 24. TxB_2_ increased in the RAL versus PI/NNRTI arm (+0.09 versus −0.02; *P* = 0.06). Baseline PGI-M was lower in the RAL arm (*P* = 0.005); no other between-arm cross-sectional differences were observed. In the PI/NNRTI arm, 24-week visceral adipose tissue change correlated with PGI-M (rho = 0.45; *P* = 0.04) and TxB_2_ (rho = 0.44; *P* = 0.005) changes, with a trend seen for PGE-M (rho = 0.41; *P* = 0.07). In an adjusted model, age ≥ 50 years (*N* = 8) was associated with increased PGE-M *(P* = 0.04). In this randomized trial, a switch to RAL did not significantly affect urinary eicosanoids over 24 weeks. In women continuing PI/NNRTI, increased visceral adipose tissue correlated with increased PGI-M and PGE-M. Older age (≥50) was associated with increased PGE-M. Relationships between aging, adiposity, ART, and eicosanoids during HIV-infection require further study.

## 1. Introduction

Fat redistribution in HIV-infected patients is associated with antiretroviral therapy (ART), including protease inhibitors (PI) and nonnucleoside reverse transcriptase inhibitors (NNRTI) [1, 2]. Central fat accumulation or lipohypertrophy may be more common in women [3, 4] and has been associated with multiple metabolic abnormalities and inflammation in HIV-infected persons on ART [5–9]. Chronic HIV infection is also associated with persistent inflammation [10], and treating HIV infection improved endothelial function in treatment-naïve subjects with low cardiovascular disease (CVD) risk [11]. Given the complex interactions between chronic HIV infection and ART and the likelihood that traditional Framingham prediction may underestimate cardiovascular risk in HIV-infected persons on ART [12, 13], novel biomarkers are needed to assess metabolic risk in HIV infection and response to interventions.

Eicosanoids are endogenous products of arachidonic acid metabolism involved in oxidant stress, inflammation, and endothelial function, all of which are important in atherosclerosis and cardiovascular disease pathogenesis [14]. Biologic properties and metabolism of eicosanoids are complex and are reviewed elsewhere [15–17]. Briefly, during cellular stress, membrane phospholipids containing arachidonic acid are subjected to nonenzymatic peroxidation by free radical species to generate a variety of biologically active oxidation products, including F_2_-isoprostanes (F_2_-IsoPs) which can cause vasoconstriction, platelet aggregation, and oxidative tissue damage. Arachidonic acid can also be released from membrane phospholipids and metabolized by oxidizing enzymes during cellular stress. Metabolism by cyclooxygenase (COX) enzymes yields a family of products termed prostaglandins (PGs), including PGE_2_, which causes vasodilation or vasoconstriction and/or vascular smooth muscle proliferation; thromboxane A_2_ (TxA_2_), which causes vasoconstriction, platelet activation, and chemotaxis; and prostacyclin (PGI_2_) which causes vasodilation and inhibits platelet aggregation and vascular smooth muscle proliferation. With the exception of F_2_-IsoPs [15], parent eicosanoids are unstable in and cannot be reliably assayed from plasma. Prostaglandin metabolites, as well as F_2_-IsoPs, are stable in urine and accurate indices of endogenous production [18–21]. The primary PGE_2_ urinary metabolite is 11-*α*-hydroxy-9,15-dioxo-2,3,4,5-tetranor-prostane-1,20-dioic acid (PGE-M), while the major metabolites used to assess TxA_2_ and PGI_2_ production are 11-dehydro-thromboxane B_2_ (TxB_2_) and 2,3-dinor-6-keto-PGF_1*α*_ (PGI-M), respectively. Urinary assays for PGE-M and F_2_-IsoP have low intra-individual variation over one year [22].

In HIV-negative populations, urinary F_2_-IsoP correlates with traditional CVD risk factors [23] and surrogate measures of CVD including brachial artery flow-mediated dilation and carotid intima media thickness [24, 25]. In obese children and adolescents, plasma F_2_-IsoP was positively correlated with visceral adipose tissue (VAT), a marker of CVD risk [26]. Cigarette smoking is also associated with higher eicosanoids [27–30]. In HIV-infected persons, cross-sectional studies to date have identified associations between higher F_2_-IsoP and lipoatrophy, lactic acidosis, virologic suppression on ART, heavy smoking, higher body mass index (BMI) and waist circumference, elevated liver transaminases and hepatitis C virus (HCV) RNA, and female sex [31–35]. Early analyses suggested higher F_2_-IsoP in persons receiving efavirenz or zidovudine [32], and lower levels in those on nevirapine compared with other ART [34] but consistent associations with specific ART drugs or classes have not been seen. It is not yet clear why F_2_-IsoP levels are consistently higher in women (HIV-infected and uninfected) than in men. A recent study of women in Haiti found higher levels of cervical COX-2 and urinary PGE-M in HIV-infected women than in uninfected women and a positive correlation between systemic PGE-M and both plasma HIV RNA and cervical COX-2 levels [36].

Raltegravir (RAL) is an HIV-1 integrase inhibitor that has not been associated with metabolic perturbations or fat redistribution during short- or long-term therapy [37–39]. A randomized, open label study was designed to assess the effects of switching from PI- or NNRTI-based ART to a RAL-based regimen in women with lipohypertrophy and suppressed HIV-1 RNA on stable therapy [40]. Adipose tissue volumes by computerized tomography (CT), anthropometrics, and fasting metabolic parameters were performed. The objectives of these secondary analyses were to determine (a) the 24-week change in F_2_-IsoP and other urinary eicosanoid metabolites and (b) correlations between 24-week changes in F_2_-IsoP, other urinary eicosanoid metabolites, and changes in VAT, the primary outcome of the parent study. We hypothesized that urinary F_2_-IsoP and other eicosanoid metabolites would decrease after 24 weeks in women switching to RAL compared to those continuing a PI/NNRTI and that these decreases would correlate with decreased VAT.

## 2. Materials and Methods

Complete methods for the parent study have been published previously [40]. Briefly, women with HIV-1 RNA <50 copies/mL, stable ART including two NRTIs (tenofovir or abacavir and emtricitabine or lamivudine) plus a PI or NNRTI, and central adiposity (waist circumference >94 cm or waist: hip > 0.88) were enrolled at five centers in North America from September 2008 to July 2010 and randomized 1 : 1 to switch their PI/NNRTI to open label RAL 400 mg twice daily (RAL arm) or continue to present ART for 24 weeks (PI/NNRTI arm). Relevant exclusion criteria included current use of metformin, thiazolidinediones, or androgen therapy, use of growth hormone or growth hormone-releasing factor in the six months prior to screening, change or initiation of lipid-lowering therapy in the three months prior to screening, and intent to significantly modify diet or exercise habits during the study. The primary endpoint of the parent trial was between-group change in percent of VAT volume 24 weeks following a switch to RAL versus continued PI or NNRTI. All study procedures were approved by the institutional review boards of the participating institutions, and all subjects provided informed consent prior to initiation of study procedures. Procedures were performed in accordance with the ethical standards of the responsible committee on human experimentation and with the Helsinki Declaration of the World Medical Association.

### 2.1. Assessments

#### 2.1.1. Anthropometric Measurements

Visceral and subcutaneous adipose tissue (VAT and SAT, resp.) volume was measured via single slice L4-L5 CT scan at weeks 0 and 24. Scans were performed locally but standardized and read centrally by a blinded reader at the Tufts University Body Composition Center. Waist, hip, and neck circumferences were performed according to AIDS Clinical Trials Group standards [41] at weeks 0, 12, and 24.

#### 2.1.2. Laboratory Assessments

Fasting (>8 hours) glucose, lipoprotein profile, high-sensitivity C reactive protein (hsCRP), and CD4+ T cell counts were assessed at weeks 0, 12, and 24. HIV-1 RNA (50 copies/mL assay sensitivity) was measured at screening and weeks 4, 8, 12, and 24. Labs were performed at the individual sites in real-time and according to local standards.

#### 2.1.3. Urinary Eicosanoids

Clean-catch urine samples were collected, and three-milliliter (mL) aliquots of urine were stored at −80°C until analysis. Samples were shipped overnight on dry ice to the Vanderbilt University Eicosanoid Core Laboratory where analyses were performed. Urinary F_2_-IsoP, TxB_2_, and PGI-M were measured using gas chromatography-negative ion chemical ionization mass spectrometry employing stable isotope dilution methodology, as described elsewhere [15, 18, 20, 42]. PGE-M was measured by liquid chromatography-mass spectroscopy (LC-MS), as previously described [19]. Results for all urinary metabolites are presented as ng/mg urinary creatinine (cr). At one study site, a freezer malfunction led to transient thawing of urine samples from nine subjects. Urinary eicosanoid results from these samples were not statistically different from the other sites, and analyses with and without data from these samples were performed (data not shown). As there were no substantive changes in the results, we report results including data from all sites.

### 2.2. Statistical Analysis

Baseline characteristics of the two randomization groups were compared using the Mann-Whitney *U* test for continuous variables and Fisher's exact test for categorical variables. Analyses also included Spearman correlations between continuous baseline and 24-week change variables. Median values and interquartile ranges (IQR) are reported for continuous variables, and percentages are reported for categorical data. Comparison of median between-group 24-week change scores for eicosanoids was performed using the Wilcoxon signed-rank test. The primary analysis was as-treated, excluding subjects who did not remain on the study regimen and/or did not have an observed primary endpoint. Generalized linear models assessed associations between eicosanoid changes and study arm, adjusting for baseline PI use, BMI, smoking status, study site, and age (≥50 versus <50 years). All statistical tests were two-sided with a nominal *P* level of 0.05. Given the exploratory nature of these analyses, we did not adjust results for multiple testing. Data analysis and management was performed using SAS 9.2 (SAS Institute, Inc., Cary, NC, USA).

## 3. Results

### 3.1. Baseline Demographics

Sixty-one women were screened and 39 enrolled in the trial. Eighteen subjects were randomized to the RAL arm and 21 to continue PI/NNRTI. One subject from each arm withdrew for reasons unrelated to the study intervention [40], leaving 37 subjects who completed the week 24 primary endpoint. Complete demographic and baseline clinical characteristics of the 37 participants included in the as-treated analysis are provided in Table 1. At baseline, the study groups were well balanced, with the exception of the PI/NNRTI arm having a higher rate of current smoking (60% versus 24%; *P* = 0.045). The median age was 43 years, BMI 32 kg/m^2^, and 75% of subjects self-identified as Black or Hispanic. Sixty-two percent of subjects were on a PI at entry (versus 38% NNRTI), and the most commonly prescribed NRTI was tenofovir (78%).

### 3.2. Baseline Urinary Eicosanoids

**Figure 1:**
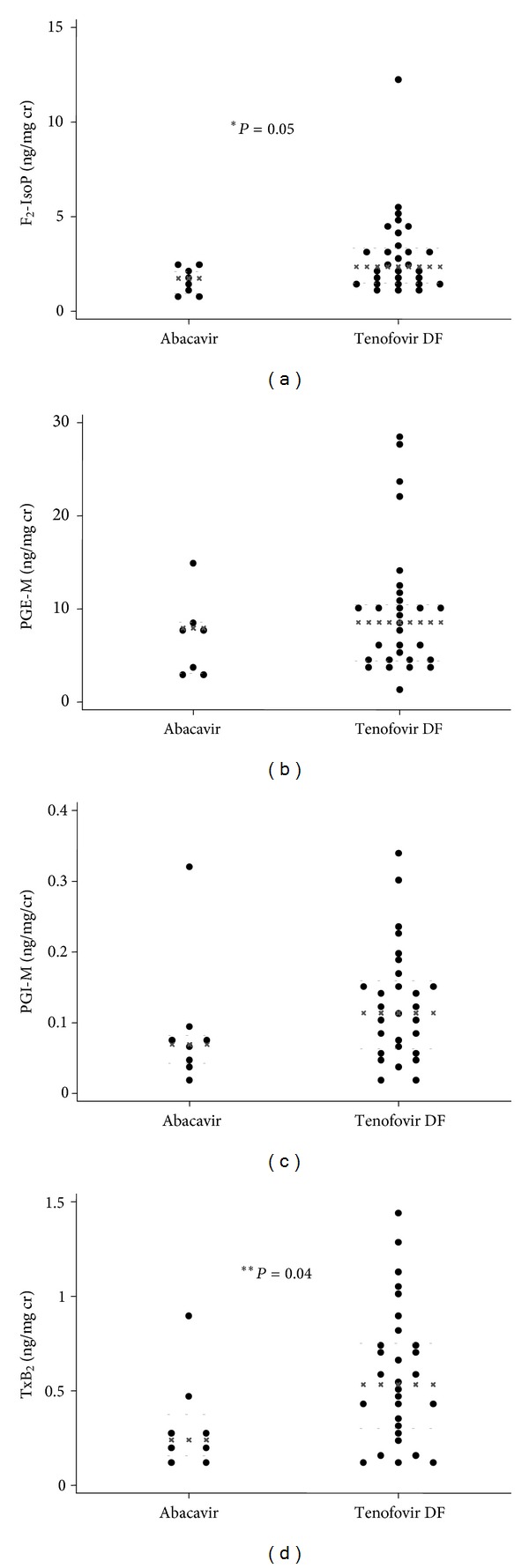
Median baseline urinary eicosanoid levels by nucleoside reverse transcriptase inhibitor use. Panel (a) shows F_2_-IsoP; Panel (b) shows PGE-M; Panel (c) shows PGI-M; Panel (d) shows TxB_2_. *P value = 0.05; **P value = 0.04. Black (x) lines indicate within-group median; grey dashed lines (-) indicate within-group 25th and 75th percentiles. F_2_-IsoPs: F_2_-isoprostanes; PGE-M: prostaglandin E_2_ metabolite; PGI-M: prostacyclin metabolite; TxB_2_: thromboxane B_2_. Units are ng/mg creatinine.

Baseline median (IQR) urinary F_2_-IsoP, PGE-M, PGI-M, and TxB_2_ (ng/mg cr) were 2.14 (1.49–3.16), 8.07 (4.47–10.56), 0.10 (0.06–0.15), and 0.46 (0.25–0.73), respectively (Table 1). When comparing study arms, baselines PGI-M, PGE-M, and TxB_2_ were all lower, and F_2_-IsoP was higher in the RAL arm (Table 1), but only PGI-M was statistically different (*P* = 0.005). Baseline PGE-M tended to be higher in current smokers (*P* = 0.1; data not shown). Eicosanoid levels also tended to differ by baseline NRTI, with women receiving abacavir having consistently lower levels (Figure 1), including statistically significantly lower F_2_-IsoP (*P* = 0.05; Figure 1(a)) and TxB_2_ (*P* = 0.04; Figure 1(d)) levels.

### 3.3. Baseline Correlations between Urinary Eicosanoids and Demographic and Metabolic Factors

Statistically significant correlations were observed at baseline with PGE-M and TxB_2_. PGE-M was positively correlated with age (rho = 0.34; *P* = 0.04) and negatively correlated with body weight (rho = −0.35; *P* = 0.03). TxB_2_ was positively correlated with VAT:SAT and VAT:total adipose tissue and negatively correlated with SAT, body weight, BMI, and hip and neck circumferences (rho = −0.37 to −0.46; *P* = 0.004 to 0.02). None of the urinary eicosanoids were correlated with fasting lipids, glucose, insulin resistance, or hsCRP in this study population at baseline (data not shown).

### 3.4. Changes in Urinary Eicosanoids by Study Arm

**Figure 2:**
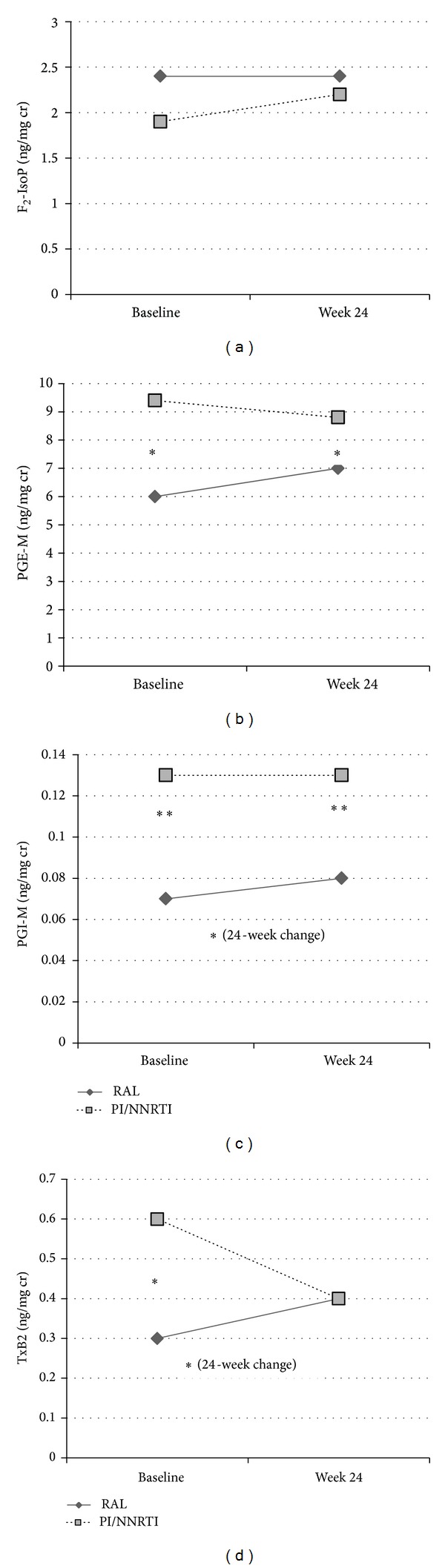
Median baseline and 24-week urinary eicosanoid levels by study arm. *Between-group *P* value <0.1–0.05; **between-group *P* value <0.05. Panel (a) shows F_2_-IsoP; Panel (b) shows PGE-M (baseline and week 24 between-group *P* = 0.08); Panel (c) shows PGI-M (baseline between-group *P* = 0.005, week 24 between-group *P* = 0.04, and between-group 24-week change *P* = 0.08); Panel (d) shows TxB_2_ (baseline between-group *P* = 0.09 and between-group 24-week change *P* = 0.06). F_2_-IsoPs: F_2_-isoprostanes; PGE-M: prostaglandin E_2_ metabolite; PGI-M: prostacyclin metabolite; TxB_2_: thromboxane B_2_. Units are ng/mg creatinine.

**Figure 3:**
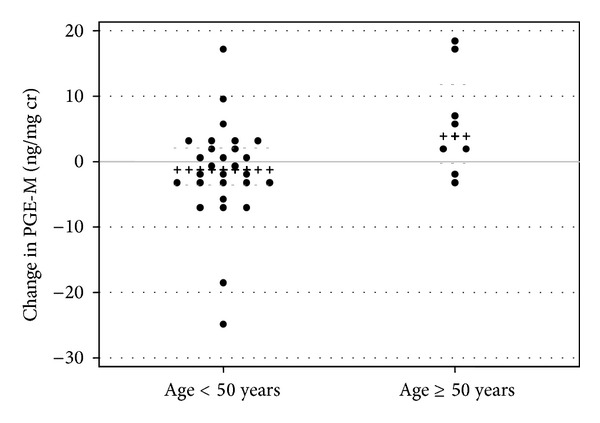
Scatterplot of 24-week change in urinary PGE-M by age < or ≥50 years. Between-group *P* = 0.05. Black (x) lines indicate within-group median; grey dashed (-) lines indicate within-group 25th and 75th percentiles. F_2_-IsoPs: F_2_-isoprostanes; PGE-M: prostaglandin E_2_ metabolite; PGI-M: prostacyclin metabolite; TxB_2_: thromboxane B_2_. Units are ng/mg creatinine.

Median 24-week urinary eicosanoid levels and changes from baseline are shown in Figure 2 and Table 2. Over 24 weeks, only PGI-M in RAL-treated subjects demonstrated a statistically significant within-group change (*P* = 0.04; Figure 2(c)). TxB_2_ increased in the RAL arm and decreased in the PI/NNRTI arm (+0.09 [−0.04, +0.13] versus −0.02 [−0.20, +0.03]; Figure 2(d)), but this difference was of borderline statistical significance (between-group *P* = 0.06). There were no other statistically significant differences between or within study arms over 24 weeks. Age ≥ 50 years at baseline was associated with an increase in PGE-M (median change +3.9 versus −1.3 in subjects <50 years of age; *P* = 0.05; Figure 3). In the PI/NNRTI arm, 24-week VAT change positively correlated with changes in PGI-M (rho = 0.45; *P* = 0.04) and TxB_2_ (rho = 0.44; *P* = 0.05), with a similar trend seen for PGE-M (rho = 0.41; *P* = 0.07). Among persons in the RAL arm, the change in PGE-M correlated with an increase in HDL cholesterol (rho = 0.56; *P* = 0.02); this correlation was not observed in the PI/NNRTI arm (rho = 0.17; *P* = 0.48). No other statistically significant correlations between lipids and urinary eicosanoids were seen. Changes in eicosanoid levels over 24 weeks were not statistically different by baseline NRTI (abacavir versus tenofovir; data not shown).

### 3.5. Adjusted Analyses of Changes in Urinary Eicosanoids

Changes in urinary eicosanoids from baseline to 24 weeks were assessed in multivariate models adjusting for study arm (RAL versus PI/NNRTI), baseline BMI, age, PI use, smoking status, and study site. Age ≥ 50 years (*N* = 8) was associated with 24-week PGE-M increase (*β* = 8.3 [95% CI 0.3, 16.3]; *P* = 0.04), independent of the covariates above (Table 3). No baseline factors were significantly associated with changes in other urinary eicosanoids (Table 3).

## 4. Discussion

In these HIV-infected women with central adiposity and suppressed HIV RNA on ART, switching PI- or NNRTI-based ART to RAL did not have significant effects on urinary eicosanoids over 24 weeks. Overall, F_2_-IsoP, PGE-M, and TxB_2_ levels were higher and PGI-M levels were lower than published levels reported in healthy adults [15, 18–20, 43, 44]. Although formal comparisons were not performed, urinary F_2_-IsoP (lower), PGE-M (higher), and TxB_2_ (higher) levels also differed from those observed in previously studied HIV-infected women who were younger and had lower BMI [33]. Of note, several of the markers differed—though not with statistical significance—at baseline between the two study arms. Given known effects of smoking on these biomarkers [27–29], this difference may have been driven in part by the significantly greater number of smokers randomized to the PI/NNRTI arm, and this may therefore have limited our capacity to identify differences in changes over time or due to RAL switch. Although baseline PGE-M tended to be higher in current smokers (*N* = 16; median [IQR] 9.9 [4.9–14.4]) than nonsmokers (*N* = 21; median [IQR] 7.9 [4.0-10.0]; *P* = 0.11), in a multivariate model, neither current smoking nor study arm was significantly associated with 24-week change in PGE-M and did not attenuate the relationship between age and change in PGE-M. Additionally, F_2_-IsoP, which is increased in HIV-infected and uninfected smokers, tended to be higher in smokers at baseline (*P* = 0.06) but was not significantly higher in the PI/NNRTI arm. Age and BMI were lower and higher in the RAL than the PI/NNRTI arm, respectively (Table 1), and though they were not statistically different, we did include these as covariates in adjusted models.

In primary analyses, women in the switch arm had significant improvements in total and LDL cholesterol but did not have a statistically significant improvement in VAT compared to women continuing an NNRTI or PI [40]. They also had a significant decrease in soluble CD14, a marker of monocyte activation [45]. A recent analysis of extensively treatment experienced, predominantly male, subjects switching enfuvirtide to RAL in France reported significant 24-week decreases in interleukin-6, D-dimer, and hsCRP [46] that were not observed in these less treatment-experienced women with central adiposity [45]. In subjects remaining on PI/NNRTI, increasing VAT was marginally correlated with increasing PGI-M and PGE-M over 24 weeks of follow-up, suggesting a relationship between these markers and central adiposity that was altered by a switch to RAL. Older age (≥50 years) at enrollment was associated with an increase in PGE-M independent of study arm, smoking status, PI use at baseline, or other factors. Urinary eicosanoids other than TxB_2_ were not significantly associated with BMI at baseline. This was unexpected given associations with F_2_-IsoP and TxB_2_ in prior cross-sectional studies of HIV-infected persons [33, 34], and this may be due to the inclusion of both males and females in prior analyses and/or the high prevalence of obesity and relative lack of normal BMI ranges in this study population.

This is the first study to prospectively assess the effects of an ART switch on urinary eicosanoid metabolites. The small sample size of our study and the imbalance of smokers in the PI/NNRTI arm likely limited our ability to detect differences between study arms. Nonetheless, intriguing trends and preliminary associations were noted. Although not routinely measured in clinical practice, eicosanoids are known markers of cardiovascular and metabolic disease risk in HIV-negative populations. In particular, F_2_-IsoP has been associated with CVD disease risk factors, hsCRP, carotid intima medial thickness, coronary artery calcium, and angiographic coronary artery obstruction [23, 24, 47]. Urinary TxB_2_ was associated with a composite clinical cardiovascular outcome [48]. PGE_2_ is a complex mediator of inflammation and vasodilation with variable effects on vascular tone depending on the tissue and prostanoid receptor [49]. Increased PGE-M has also been associated with malignancies in HIV-negative populations [50–53], and urinary PGE-M correlated with plasma HIV RNA and cervical COX-2 levels in a recent study of Haitian women [36].

It was notable that baseline F_2_-IsoP and TxB_2_ were lower in subjects on abacavir. Abacavir exposure has been associated with increased cardiovascular risk in HIV-infected persons [54], but the association has not been consistent [55]. Although potential mechanism(s) are not clearly defined, recent studies have reported abnormal platelet reactivity with abacavir [56, 57]. To our knowledge, TxB_2_ has not been assessed in these or other studies. Lower F_2_-IsoP in these abacavir-treated subjects and in a prior cross-sectional analysis of men and women [33] suggests that if there is excess cardiovascular risk due to abacavir, it is independent of lipid peroxidation-related pathways; prospective studies would be needed to determine this. Due to providers' knowledge of potential cardiovascular risk with abacavir and risk of renal toxicity with tenofovir DF, it is also possible that abacavir use is simply a marker of some other unmeasured factors associated with risk for these conditions and lower urinary eicosanoid levels.

In addition to the qualifications above, this analysis has other limitations that should be noted. Use of aspirin or nonsteroidal anti-inflammatory drugs (NSAID) was not an exclusion criterion for the parent study, and detailed information on dosing was not collected. Given the even distribution of NSAID use across study arms (Table 1), we did not adjust for this variable in multivariate models and do not believe it would explain differential associations within or between groups. Neither menopausal status nor sex hormone levels were ascertained as part of this study. Based on data from HIV-infected women [58], the age distribution of our population suggests the majority of subjects were pre- or perimenopausal. The association between age and PGE-M may have been due to postmenopausal changes in the older women (≥50 years). The number of older women included in this study was small. Platelet reactivity assays were not performed, so relationships between eicosanoids and platelet function cannot be determined. CT scan was used to assess SAT, so we were unable to fully assess peripheral (limb) lipoatrophy. Finally, this analysis may have been too small and/or of insufficient duration of follow-up to detect meaningful changes in eicosanoids. Additional studies and longer-term follow-up in HIV-infected persons are needed to further elucidate the role of eicosanoids in metabolic and other aging-related comorbidities and determine their role as useful clinical biomarkers.

## Figures and Tables

**Table 1:** Baseline characteristics of subjects completing 24 weeks of follow-up and included in eicosanoid analyses, total and by study arm.

	Total (*N* = 37)		RAL (*N* = 17)		PI/NNRTI (*N* = 20)	
Ethnicity						
African-American	22 (59)		9 (53)		13 (65)	
Hispanic	6 (16)		4 (24)		2 (10)	
White	8 (22)		3 (18)		5 (25)	
Asian	1 (3)		1 (6)		0 (0)	
Age in years-median (range)	43 (26–57)		41 (26–51)		46 (31–57)	
Weight (kg)	81.8 (73.9–105.0)		88.7 (81.0–105.0)		77.7 (71.7–97.0)	
BMI (kg/m^2^)	32.0 (28.0–36.5)		34.7 (28.8–37.6)		30.4 (27.7–35.4)	
VAT (cm^2^)	138 (100–154)		145 (105–154)		138 (93–154)	
SAT (cm^2^)	432 (343–605)		450 (381–687)		420 (342–587)	
VAT : SAT	0.25 (0.21–0.38)		0.25 (0.22–0.36)		0.25 (0.20–0.42)	
Total cholesterol (mg/dL)	188.0 (162.0–214.0)		179.0 (162.0–206.0)		199.0 (164.5–221.5)	
Triglycerides (mg/dL)^c^	118.0 (92.0–152.0)		116.0 (85.0–144.0)		129.0 (101.0–176.0)	
LDL (mg/dL)	115.8 (93.0–128.0)		113.0 (103.0–123.0)		116 (89.0–138.1)	
HDL (mg/dL)	49.0 (40.0–57.0)		47.6 (40.2–57.0)		49.1 (39.0–55.0)	
Glucose (mg/dL)	87.0 (78.0–94.0)		84.0 (78.0–93.0)		88.5 (80.0–97.5)	
Tobacco Use (Current)	16 (43)		4 (24)^a^		12 (60)^a^	
Daily anti-inflammatory use^b^	14 (38)		7 (41)		7 (35)	
CD4 count (cells/uL)	558 (422–747)		563 (447–747)		553 (354–770)	
Baseline ART regimen						
PI	23 (62)		11 (65)		12 (60)	
NNRTI	14 (38)		6 (35)		8 (40)	
NRTI						
Abacavir	8 (22)		3 (18)		5 (25)	
Tenofovir	29 (78)		14 (82)		15 (75)	

Urinary eicosanoids (ng/mg cr)	Median (IQR)	Mean (SD)	Median (IQR)	Mean (SD)	Median (IQR)	Mean (SD)

F_2_-IsoP	2.14 (1.49–3.16)	2.70 (2.09)	2.41 (1.96–3.04)	2.59 (1.14)	1.89 (1.35–3.77)	2.79 (2.67)
PGE-M (*N* = 36)	8.07 (4.47–10.56)	9.35 (6.77)	5.95 (4.25–9.52)	7.35 (6.07)	9.37 (5.36–13.48)	10.80 (7.10)
PGI-M (*N* = 36)	0.10 (0.06–0.15)	0.12 (0.08)	0.07 (0.05–0.10)^c^	0.08 (0.04)	0.13 (0.08–0.21)^c^	0.15 (0.09)
TxB_2_	0.46 (0.25–0.73)	0.53 (0.36)	0.34 (0.14–0.70)	0.45 (0.37)	0.55 (0.29–0.77)	0.60 (0.34)

Values shown are *N* (percentage), median (interquartile range [IQR]), or mean (standard deviation [SD]) except where noted.

^a^Fisher's exact *P* = 0.045 for immediate versus delayed arm. ^b^Seven women in each arm reported daily anti-inflammatory medication use: six in each arm reported non-steroidal anti-inflammatory drug use; one in the RAL arm reported taking celecoxib, and one in the PI/NNRTI arm reported taking aspirin 81 mg. ^c^Mann-Whitney *UP* = 0.005 for RAL versus PI/NNRTI arm.

Abbreviations: ART = antiretroviral therapy; BMI = body mass index; F_2_-IsoP = F_2_-isoprostanes; HDL = high density lipoprotein cholesterol; LDL = low density lipoprotein cholesterol; NRTI = nucleoside reverse transcriptase inhibitor; NNRTI = non-NRTI; PI = protease inhibitor; PGE-M = prostaglandin E_2_ metabolite; PGI-M = prostacyclin metabolite; RAL = raltegravir; SAT = subcutaneous adipose tissue; TxB_2_= thromboxane B_2_; VAT = visceral adipose tissue.

**Table 2:** Urinary eicosanoid levels- absolute and 24-week changes, by study arm.

	RAL	Within-group *P*	PI/NNRTI	Within-group *P*	Between-group *P*
F_2_-IsoP					
Week 0	2.41 (1.96, 3.04)		1.89 (1.35, 3.77)		0.28
Week 24	2.39 (1.55, 2.65)		2.20 (1.17, 3.10)		0.96
Week 24 change	−0.12 (−0.3, +0.08)	0.13	−0.13 (−0.62, +0.60)	0.70	0.82
PGE-M					
Week 0	5.95 (4.25, 9.52)		9.37 (5.36, 13.48)		0.08
Week 24	6.96 (2.14, 8.51)		8.84 (5.77, 13.26)		0.08
Week 24 change	+1.07 (−3.26, +3.08)	0.82	−1.25 (−3.29, +3.23)	0.81	0.77
PGI-M					
Week 0	0.07 (0.05, 0.10)		0.13 (0.08, 0.21)		0.005
Week 24	0.08 (0.06, 0.13)		0.13 (0.10, 0.18)		0.04
Week 24 change	+0.02 (−0.002, +0.05)	0.04	−0.004 (−0.04, +0.03)	0.62	0.08
TxB_2_					
Week 0	0.34 (0.14, 0.70)		0.55 (0.29, 0.77)		0.09
Week 24	0.44 (0.27, 0.64)		0.44 (0.35, 0.61)		0.56
Week 24 change	+0.10 (−0.04, +0.13)	0.22	−0.02 (−0.20, +0.03)	0.25	0.07

Values shown are median (interquartile range).

F_2_-IsoP = F_2_-isoprostanes; NNRTI = non-nucleoside reverse transcriptase inhibitor; PI = protease inhibitor; PGE-M = prostaglandin E_2_ metabolite; PGI-M = prostacyclin metabolite; RAL = raltegravir; TxB_2_ = thromboxane B_2_.

**Table 3:** Multivariate generalized linear models of predictors of 24-week eicosanoid changes.

Covariate	F_2_-IsoP		PGE-M		PGI-M		TxB_2_	
	*β* (95% CI)	*P*	*β* (95% CI)	*P*	*β* (95% CI)	*P*	*β* (95% CI)	*P*
Study arm (RAL versus PI/NNRTI)	0.25 (−0.79, 1.30)	0.63	1.6 (−4.5, 7.7)	0.60	0.04 (−0.02, 0.09)	0.19	0.12 (−0.02, 0.26)	0.10
PI use at baseline (yes versus no)	0.46 (−0.54, 1.46)	0.35	−3.9 (−9.6, 1.8)	0.17	0.004 (−0.05, 0.05)	0.85	0.06 (−0.07, 0.20)	0.35
Age ≥ 50 years (versus <50)	0.73 (−0.66, 2.12)	0.29	8.3 (0.3, 16.3)	0.04	0.02 (−0.06, 0.10)	0.59	−0.02 (−0.21, 0.17)	0.81
Current smoking (yes versus no)	−0.37 (−1.54, 0.81)	0.53	0.9 (−6.0, 7.9)	0.78	−0.01 (−0.07, 0.05)	0.69	0.11 (−0.05, 0.27)	0.18
Baseline BMI	0.02 (−0.06, 0.09)	0.63	0.3 (−0.1, 0.7)	0.13	0.0005 (−0.003, 0.004)	0.80	0.007 (−0.003, 0.02)	0.16

BMI = body mass index; F_2_-IsoP = F_2_-isoprostanes; NNRTI = non-nucleoside reverse transcriptase inhibitor; PI = protease inhibitor; PGE-M = prostaglandin E_2_ metabolite; PGI-M = prostacyclin metabolite; RAL = raltegravir; TxB_2_ = thromboxane B_2_.

## References

[1] Carr A (2003). HIV lipodystrophy: risk factors, pathogenesis, diagnosis and management. *AIDS*.

[2] Haubrich RH, Riddler SA, Dirienzo AG (2009). Metabolic outcomes in a randomized trial of nucleoside, nonnucleoside and protease inhibitor-sparing regimens for initial HIV treatment. *AIDS*.

[3] Health KV, Chan KJ, Singer J, O’Shaughnessy MV, Montaner JSG, Hogg RS (2002). Incidence of morphological and lipid abnormalities: gender and treatment differentials after initiation of first antiretroviral therapy. *International Journal of Epidemiology*.

[4] Cabrero E, Griffa L, Burgos A (2010). Prevalence and impact of body physical changes in HIV patients treated with highly active antiretroviral therapy: results from a study on patient and physician perceptions. *AIDS Patient Care and STDs*.

[5] Currier J, Scherzer R, Bacchetti P (2008). Regional adipose tissue and lipid and lipoprotein levels in HIV-infected women. *Journal of Acquired Immune Deficiency Syndromes*.

[6] Wohl D, Scherzer R, Heymsfield S (2008). The associations of regional adipose tissue with lipid and lipoprotein levels in HIV-infected men. *Journal of Acquired Immune Deficiency Syndromes*.

[7] Grunfeld C, Kotler DP, Arnett DK (2008). Contribution of metabolic and anthropometric abnormalities to cardiovascular disease risk factors. *Circulation*.

[8] Falutz J (2011). HIV infection, body composition changes and related metabolic complications: contributing factors and evolving management strategies. *Current Opinion in Clinical Nutrition and Metabolic Care*.

[9] Johnson JA, Albu JB, Engelson ES (2004). Increased systemic and adipose tissue cytokines in patients with HIV-associated lipodystrophy. *American Journal of Physiology, Endocrinology and Metabolism*.

[10] Neuhaus J, Jacobs DR, Baker JV (2010). Markers of inflammation, coagulation, and renal function are elevated in adults with HIV infection. *Journal of Infectious Diseases*.

[11] Torriani FJ, Komarow L, Parker RA (2008). Endothelial function in human immunodeficiency virus-infected antiretroviral-naive subjects before and after starting potent antiretroviral therapy. The ACTG (AIDS Clinical Trials Group) study 5152s. *Journal of the American College of Cardiology*.

[12] Law MG, Friis-Møller N, El-Sadr WM (2006). The use of the Framingham equation to predict myocardial infarctions in HIV-infected patients: comparison with observed events in the D:A:D Study. *HIV Medicine*.

[13] Parra S, Coll B, Aragonés G (2010). Nonconcordance between subclinical atherosclerosis and the calculated Framingham risk score in HIV-infected patients: relationships with serum markers of oxidation and inflammation. *HIV Medicine*.

[14] Morrow JD (2005). Quantification of isoprostanes as indices of oxidant stress and the risk of atherosclerosis in humans. *Arteriosclerosis, Thrombosis, and Vascular Biology*.

[15] Milne GL, Yin H, Brooks JD, Sanchez S, Jackson Roberts L, Morrow JD (2007). Quantification of F2-isoprostanes in biological fluids and tissues as a measure of oxidant stress. *Methods in Enzymology*.

[16] Milne GL, Morrow JD (2006). Isoprostanes and related compounds: update 2006. *Antioxidants and Redox Signaling*.

[17] Pratico D, Dogne JM (2009). Vascular biology of eicosanoids and atherogenesis. *Expert Review of Cardiovascular Therapy*.

[18] Morrow JD, Minton TA (1993). Improved assay for the quantification of 11-dehydrothromboxane B2 by gas chromatography-mass spectrometry. *Journal of Chromatography B: Biomedical Sciences and Applications*.

[19] Murphey LJ, Williams MK, Sanchez SC (2004). Quantification of the major urinary metabolite of PGE 2 by a liquid chromatographic/mass spectrometric assay: determination of cyclooxygenase-specific PGE 2 synthesis in healthy humans and those with lung cancer. *Analytical Biochemistry*.

[20] Daniel VC, Minton TA, Brown NJ, Nadeau JH, Morrow JD (1994). Simplified assay for the quantification of 2,3-dinor-6-ketoprostaglandin F1*α* by gas chromatography-mass spectrometry. *Journal of Chromatography B: Biomedical Sciences and Applications*.

[21] Dorjgochoo T, Gao Y-T, Chow W-H (2012). Major metabolite of F2-isoprostane in urine may be a more sensitive biomarker of oxidative stress than isoprostane itself. *American Journal of Clinical Nutrition*.

[22] Wu X, Cai H, Xiang Y-B (2010). Intra-person variation of urinary biomarkers of oxidative stress and inflammation. *Cancer Epidemiology Biomarkers and Prevention*.

[23] Schwedhelm E, Bartling A, Lenzen H (2004). Urinary 8-iso-prostaglandin F2*α* as a risk marker in patients with coronary heart disease: a matched case-control study. *Circulation*.

[24] Basarici I, Altekin RE, Demir I, Yilmaz H (2007). Associations of isoprostanes-related oxidative stress with surrogate subclinical indices and angiographic measures of atherosclerosis. *Coronary Artery Disease*.

[25] Martino F, Loffredo L, Carnevale R (2008). Oxidative stress is associated with arterial dysfunction and enhanced intima-media thickness in children with hypercholesterolemia: the potential role of nicotinamide-adenine dinucleotide phosphate oxidase. *Pediatrics*.

[26] Araki S, Dobashi K, Yamamoto Y, Asayama K, Kusuhara K (2010). Increased plasma isoprostane is associated with visceral fat, high molecular weight adiponectin, and metabolic complications in obese children. *European Journal of Pediatrics*.

[27] Gross ND, Boyle JO, Morrow JD (2005). Levels of prostaglandin E metabolite, the major urinary metabolite of prostaglandin E_2_, are increased in smokers. *Clinical Cancer Research*.

[28] Morrow JD, Frei B, Longmire AW (1995). Increase in circulating products of lipid peroxidation (F_2_-isoprostanes) in smokers: Smoking as a cause of oxidative damage. *The New England Journal of Medicine*.

[29] Rångemark C, Benthin G, Granström EF, Persson L, Winell S, Wennmalm Å (1992). Tobacco use and urinary excretion of thromboxane A_2_ and prostacyclin metabolites in women stratified by age. *Circulation*.

[30] McAdam BF, Byrne D, Morrow JD, Oates JA (2005). Contribution of cyclooxygenase-2 to elevated biosynthesis of thromboxane A_2_ and prostacyclin in cigarette smokers. *Circulation*.

[31] McComsey GA, Morrow JD (2003). Lipid oxidative markers are significantly increased in lipoatrophy but not in sustained asymptomatic hyperlactatemia. *Journal of Acquired Immune Deficiency Syndromes*.

[32] Hulgan T, Morrow J, D’Aquila RT (2003). Oxidant stress is increased during treatment of human immunodeficiency virus infection. *Clinical Infectious Diseases*.

[33] Boger MS, Bian A, Shintani A (2012). Sex differences in urinary biomarkers of vascular and endothelial function in HIV-infected persons receiving antiretroviral therapy. *Antiviral Therapy*.

[34] Redhage LA, Shintani A, Haas DW (2009). Clinical factors associated with plasma F_2_-isoprostane levels in HIV-infected adults. *HIV Clinical Trials*.

[35] Glesby MJ, Hoover DR, Raiszadeh F (2009). Oxidant stress in HIV-infected women from the Women’s Interagency HIV Study. *Antiviral Therapy*.

[36] Fitzgerald DW, Bezak K, Ocheretina O (2012). The effect of HIV and HPV coinfection on cervical COX-2 expression and systemic prostaglandin E_2_ levels. *Cancer Prevention Research*.

[37] Eron JJ, Cooper DA, Steigbigel RT (2013). Efficacy and safety of raltegravir for treatment of HIV for 5 years in the BENCHMRK studies: final results of two randomised, placebo-controlled trials. *The Lancet Infectious Diseases*.

[38] Lennox JL, DeJesus E, Lazzarin A (2009). Safety and efficacy of raltegravir-based versus efavirenz-based combination therapy in treatment-naive patients with HIV-1 infection: a multicentre, double-blind randomised controlled trial. *The Lancet*.

[39] Eron JJ, Young B, Cooper DA (2010). Switch to a raltegravir-based regimen versus continuation of a lopinavir-ritonavir-based regimen in stable HIV-infected patients with suppressed viraemia (SWITCHMRK 1 and 2): two multicentre, double-blind, randomised controlled trials. *The Lancet*.

[40] Lake JE, McComsey GA, Hulgan TM (2012). A randomized trial of raltegravir replacement for protease inhibitor or non-nucleoside reverse transcriptase inhibitor in HIV-infected women with lipohypertrophy. *AIDS Patient Care and STDs*.

[42] Milne GL, Gao B, Terry ES, Zackert WE, Sanchez SC (2013). Measurement of F_2_-isoprostanes and isofurans using gas chromatography-mass spectrometry. *Free Radical Biology and Medicine*.

[43] Rho YH, Chung CP, Oeser A (2010). Interaction between oxidative stress and high-density lipoprotein cholesterol is associated with severity of coronary artery calcification in rheumatoid arthritis. *Arthritis Care and Research*.

[44] Avalos I, Chung CP, Oeser A (2007). Aspirin therapy and thromboxane biosynthesis in systemic lupus erythematosus. *Lupus*.

[45] Lake JE, McComsey GA, Hulgan T, Wanke C, Mangili A Soluble CD14 declines in virologically suppressed women switching from protease inhibitor or NNRTI to raltegravir: the women, integrase, and fat accumulation trial.

[46] Silva EF, Charreau I, Gourmel B (2013). Decreases in inflammatory and coagulation biomarkers levels in HIV-infected patients switching from enfuvirtide to raltegravir: ANRS 138 substudy. *Journal of Infectious Diseases*.

[47] Gross M, Steffes M, Jacobs DR (2005). Plasma F_2_-isoprostanes and coronary artery calcification: the CARDIA study. *Clinical Chemistry*.

[48] Eikelboom JW, Hirsh J, Weitz JI, Johnston M, Yi Q, Yusuf S (2002). Aspirin-resistant thromboxane biosynthesis and the risk of myocardial infarction, stroke, or cardiovascular death in patients at high risk for cardiovascular events. *Circulation*.

[49] Breyer RM, Bagdassarian CK, Myers SA, Breyer MD (2001). Prostanoid receptors: subtypes and signaling. *Annual Review of Pharmacology and Toxicology*.

[50] Kim S, Taylor JA, Milne GL, Sandler DP (2013). Association between urinary prostaglandin E_2_ metabolite and breast cancer risk: a prospective, case-cohort study of postmenopausal women. *Cancer Prevention Research*.

[51] Shrubsole MJ, Cai Q, Wen W (2012). Urinary prostaglandin E_2_ metabolite and risk for colorectal adenoma. *Cancer Prevention Research*.

[52] Dong LM, Shu X-O, Gao Y-T (2009). Urinary prostaglandin E_2_ metabolite and gastric cancer risk in the Shanghai women’s health study. *Cancer Epidemiology Biomarkers and Prevention*.

[53] Kekatpure VD, Boyle JO, Zhou XK (2009). Elevated levels of urinary prostaglandin E metabolite indicate a poor prognosis in ever smoker head and neck squamous cell carcinoma patients. *Cancer Prevention Research*.

[54] Sabin CA, Worm SW, Weber R, Reiss P, El-Sadr W (2008). Use of nucleoside reverse transcriptase inhibitors and risk of myocardial infarction in HIV-infected patients enrolled in the D:A:D study: a multi-cohort collaboration. *The Lancet*.

[55] Ribaudo HJ, Benson CA, Zheng Y (2011). No risk of myocardial infarction associated with initial antiretroviral treatment containing abacavir: short and long-term results from ACTG A5001/ALLRT. *Clinical Infectious Diseases*.

[56] Satchell CS, O’Halloran JA, Cotter AG (2011). Increased platelet reactivity in HIV-1-infected patients receiving abacavir-containing antiretroviral therapy. *Journal of Infectious Diseases*.

[57] Falcinelli E, Francisci D, Belfiori B (2013). In vivo platelet activation and platelet hyperreactivity in abacavir-treated HIV-infected patients. *Thrombosis and Haemostasis*.

[58] Schoembaum EE, Hartel D, Lo Y (2005). HIV infection, drug use, and onset of natural menopause. *Clinical Infectious Diseases*.

